# Cost of diabetes care in out-patient clinics of Karachi, Pakistan

**DOI:** 10.1186/1472-6963-7-189

**Published:** 2007-11-21

**Authors:** Liaquat A Khowaja, Ali K Khuwaja, Peter Cosgrove

**Affiliations:** 1 Community Health Sciences Department, Aga Khan University, Karachi, Pakistan; 2 Family Medicine Department, Aga Khan University, Karachi, Pakistan

## Abstract

**Background:**

Diabetes Mellitus (DM) is a growing epidemic and the cost of treating diabetes is largely increasing. The objective of this study was to estimate the cost-of-illness of DM among attendees of out-patient clinics in Karachi, Pakistan. This is the first study conducted from a societal perspective to estimate the cost of managing diabetes in Pakistan.

**Methods:**

A prevalence-based 'Cost-of-Illness' study for diabetes care was conducted in six different out-patient clinics of Karachi, Pakistan from July to September 2006. A pre-tested questionnaire was administered to collect the data from 345 randomly selected persons with diabetes.

**Results:**

The annual mean direct cost for each person with diabetes was estimated to be Pakistani rupees 11,580 (US$ 197). Medicines accounted for the largest share of direct cost (46%), followed by laboratory investigations (32%). We found that increased age, the number of complications and longer duration of the disease significantly increase the burden of cost on society (p < 0.001). Comparing cost with family income it was found that the poorest segment of society is spending 18% of total family income on diabetes care.

**Conclusion:**

This study concluded that substantial expenditure is incurred by people with diabetes; with the implication that resources could be saved by prevention, earlier detection and a reduction in diabetes co-morbidities and complications through improved diabetes care. Large scale and cost-effective prevention programs need to be initiated to maximise health gains and to reverse the advance of this epidemic.

## Background

Diabetes Mellitus (DM) is a chronic and potentially disabling disease. It is a major and growing threat to global public health. The biggest impact of the disease is on adults of working age; particularly in developing countries [1]. The prevalence of diabetes and its adverse health effects have risen more rapidly in South Asia than in any other region of the world [2]. Diabetes significantly adds to the burden of preventable diseases and leads to economic losses that stem from high cost of care and loss of productivity [3]. In South Asia the majority of people live on or below the poverty line and having lack of access to healthcare services, lack of national welfare schemes and provision of health insurance for the poor population. The poor people can not afford to pay for healthcare services, hence they are diagnosed late with diabetes resulting in acute and chronic complications [4]. The prevalence of diabetes in Pakistan in the age group 20–79 years is 6.2 million [5]; which indicates that over 11% of the adult population are suffering from DM. Moreover, a similar proportion of the population in Pakistan have impaired glucose tolerance test (IGT) [6]; which is expected to rise more rapidly in future.

DM is associated with a large variety of complications and a greater risk of all manifestations of atherosclerosis [7]. Once diabetes develops, it is a costly disease to manage because of its chronic nature and severity of complications [8]. Over 70% of diabetes related cost is attributed to its complications, particularly for macro-vascular diseases which most commonly occur in type 2 diabetics [4]. In a multi-centre study from Pakistan Khuwaja et al, reported that a substantial number of people with diabetes were suffering with macro-vascular complications [7]. In India, Ramachandran and colleagues reported that diabetes is more common in the higher socio economic class while diabetes complications are more prevalent among the lower socio economic class [9]. When diabetes and its complications affect the principal breadwinner; the choice between healthcare expenses and food or clothing can trap the whole family in a downward spiral of worsening poverty and health [10].

Globally the direct healthcare cost of diabetes for people in the 20 to 79 age group is estimated to be at least $153 billion annually [11]. Diabetes alone claims on average around 8% of total health care budgets in developed countries [12]. The healthcare cost of one diabetic patient varies hugely between countries e.g. from US$ 13 in Bangladesh to US$ 11,157 in the USA per year [13]. The economic aspects of diabetes and its care continue to attract attention as the world diabetes epidemic progresses and health care systems remain under pressure to accomplish more and more within constrained resources [11]. The economic burden resulting from diabetes is a major concern for many developed and developing countries. Medical cost is two to three fold higher [14] and the economic cost is two to five fold higher in people who have diabetes compared to those without diabetes [15]. The increasing healthcare cost is limiting healthcare resources all over the world especially in developing countries. There is dire need of urgent attention and action to initiate and augment prevention programs against this disease to reduce the cost burden to society. The governmental and non-governmental organizations NGO), diabetes associations, health professionals and persons with diabetes themselves need to be aware of the current and future economic impact of this disease on the individual, family, society and healthcare institutions [11].

Studies on the cost of diabetes are scanty in the South Asian region. In Pakistan no such study has been documented to calculate the cost of diabetes. In this era of scarce resources and rising cost, it is critical to have an understanding of the economic aspect of diabetes in order to develop and implement sound public health and prevention policies. This cost-of-illness study aimed to estimate the economic burden of diabetes in Karachi, Pakistan from the societal perspective.

## Methods

A prevalence-based "Cost of Illness" study design was used to estimate the cost of diabetes care in different out-patient clinics of Karachi, Pakistan. Karachi, a city of more than 15 million inhabitants, is the largest metropolitan city and is considered a leading industrial, commercial and financial hub of Pakistan [16].

At the core, cost of illness estimates represent a descriptive economic method [17] which is most often used to estimate the cost of a particular disease. This study was carried out in six different out-patient clinics at three selected sites in Karachi representing public, private and NGO provision. From each setting, two clinics including diabetes management and family practice/general medicine/internal medicine were selected. Further, it was assumed that all levels of socio-economic status (SES) would be captured in these clinics. Diabetic subjects who fulfilled the eligibility criteria were selected using systematic random sampling technique from the six out-patient clinics at the three sites i.e. two clinics from each site. The data was collected from July 2006 to August 2006 by trained data collectors.

A total 353 eligible subjects were approached based on a proportionate attendance of patients in the different studied clinics. Informed consent was taken prior to conducting the interview from each study subject. We intended to approach all the persons with diabetes (type 1 and type 2) however in random selection no person was found with type 1 diabetes; hence all the study subjects were with type 2 diabetes. From all randomly selected subjects, 345 (98% response rate) were found willing to participate in the study. The subjects excluded from the study were patients with pregnancy; people less than 20 years or more than 60 years of age; and those who were diagnosed as diabetic in the current visit (i.e. newly diagnosed). Taken to be the most productive years, subjects in the age range 20–60 years were recruited in the study. A structured pre-tested questionnaire was used to collect the information on socio-demographic and clinical characteristics (see additional file 1). These variables were also checked and verified from the medical records of patients. All subjects were asked about direct and indirect health care costs. Some costs were imputed to reflect opportunity cost. Indirect healthcare cost was calculated using the human capital approach; here average wage rates/replacement costs were used to impute values. The human capital approach considers the value of lost productivity as a result of disability and premature death, using lost earnings as a surrogate for the impact that premature death and disability had on individuals and society [18]. Data on indirect costs covered in this study include the cost for loss of productivity and loss of time (during travelling, waiting in clinic and consultation). The cost for lost productivity has been estimated (for office job employees, businessmen, laborers and housewives) by using respective wage rate per hour. The Pharmacy guide [19] for the year 2004 was used to calculate the cost for medicines. SES was obtained using criteria (based on household items) of National Health Survey of Pakistan (NHSP) [20]. The wage rate of housewives was estimated using minimum wage rate of Pakistan (Rs. 16.7/- per hour) [21]. Costs were measured in Pakistani Rupee; to express costs in terms of international currency we applied a rate of Rs. 60 per US$.

### Perspective

Perspective is important because it determines the types of costs to be included in the study. This cost of illness study has been conducted from the societal perspective. Adoption of the societal perspective facilitates policies aimed at maximizing the welfare gains to society, or minimizing the losses.

### Ethical approval

Ethical approval was taken from the Ethical Review Committee (ERC) of the Aga Khan University (AKU). Written permission was sought from all clinical administrators and written informed consent was taken from individual study subjects. All literate individual subjects read the consent paper by themselves and signed. For the illiterate subjects, the data collector read and translated the consent paper to them and if they agreed, their thumb impression or sign was taken.

### Data management and analysis

Data were validated after double entry and then analyzed on the Statistical Package for the Social Sciences (SPSS) version 14. Mean and standard deviations were calculated for all the cost variables. For association of outcome with independent variables, univariate analysis was carried out by 'Kruskal Wallis' and 'Mann Whitney U' tests with a significance level of 5%. For this analysis, cost variables were categorized as appropriate and mean cost was compared between the categories of independent variables. The costs for subsidized services at public and charitable healthcare providers were imputed using the approximate average cost for respective services. Indirect healthcare costs for study subjects and their accompanying persons were calculated using the human capital approach.

## Results

### Socio-demographic and clinical characteristics

A total of 345 persons with diabetes were interviewed with mean (SD) age 48.12 (9.12). A Majority (64%) of study subjects were female and only 18% of subjects had graduated or having higher level of education. By occupation, the majority (55%) were housewives and only (17%) were employed in office job or having their own business. Of the total, about (48%) had a household income ranges between Rs. 5,001 – 20,000. Using NHSP criteria, we found that about 60% of households would be of middle SES, with 7% and 33% of households categorized as low and high SES respectively. A larger group of the respondents (73%) were treating their diabetes with oral medication while (21%) and (6%) were treating their diabetes with insulin and lifestyle modification respectively. A majority of subjects (72%) had one or more diabetes related co-morbidity (Table 1).

**Table 1:** Distribution of socio-demographic and clinical characteristics among persons with diabetes attended different diabetes clinics in Karachi, Pakistan (n = 345)

**Characteristics**	**Number**	**Percentages**
**Age**		
20 – 40 years	90	26.1
41 – 50 years	124	35.9
51 – 60 years	131	38.0
**Sex**		
Male	126	36.5
Female	219	63.5
**Education**		
Illiterate	102	29.6
Up to primary	74	21.4
Secondary to intermediate	107	31.0
Graduate and above	62	18.0
**Employment status**		
Jobless	40	11.6
Laborer	56	16.2
Housewife	190	55.1
Office job/businessman	59	17.1
**Monthly household income**		
Up to 5000	82	23.8
5001 – 20000	165	47.8
> 20000	98	28.4
**Socioeconomic status (SES)**		
Low SES	23	6.7
Middle SES	208	60.3
High SES	114	33.0
**Duration of diabetes**		
1–5 years	185	53.6
6–10 years	87	25.2
>10 years	73	21.2
**Mode of treatment**		
Lifestyle modification	20	5.8
Oral medication	252	73.0
Insulin	73	21.2
**Co-morbidities**		
No co-morbidity	97	28.1
1 co-morbidity	81	23.5
>1 co-morbidity	167	48.4

We focused on the use of resources for the one out-patient visit including costs of consultation, investigation, medication, travel and food costs. We found that on average each patient visited every second month, hence this cost is calculated for one visit (two months approximately). More specifically, to estimate costs subjects were asked the number of times they visited the doctor per year. A more detailed response was required for medications so the last month was used; which included questions on dose, frequency and expenditure. All other costs were based on the current visit and extrapolated.

### Direct and indirect cost of diabetes care

In the current visit the mean cost for consultation with a physician, laboratory investigations and medicines came to Rs. 205, 516 and 748 respectively. The mean cost for travel and food incurred was Rs. 120 and 33 respectively. From the total diabetes cost components, the cost for medicine represents the largest share (46%), followed by laboratory cost (32%). The unit cost has been estimated in terms of mean cost per visit for each of the cost variable. Total mean direct cost borne by the person with diabetes and/or his/her family is estimated to be Rs. 1,930 in the current visit. The value for lost productivity was calculated based on the average earnings of those whose productivity was forgone. Excluding unemployed persons, the mean lost productivity by study subjects and their attendants was Rs. 113 and 208 respectively and their mean time lost was 3 hours. We found that overall the mean economic cost borne by each person with diabetes and/or his/her family came to Rs. 2,070/- for each visit. Adjusting this cost to monthly and yearly basis, it amounts to Rs. 1,035/- and 12,420/- respectively (data not shown) (Table 2).

**Table 2:** Direct and indirect cost in Pakistani Rupee of studied persons with diabetes per visit

**Variables**	**Mean (SD)**
**Direct cost**	
Consultation cost	205.1 (277.3)
Investigations cost	515.8 (809.7)
Medicine cost	748.0 (682.9)
Travel cost	119.7 (232.6)
Food on way to clinic cost	33.2 (56.6)
Subsidized consultation cost for public hospital based clinics and welfare clinic* N**	201.7 (- -)
Subsidized investigation cost for public hospital based clinics and welfare clinic* N**	494.0 (- -)
**Indirect cost**	
**Lost time (patients)**	
Office employees/businessmen	3.5 (4.0)
Laborers	3.2 (2.2)
Housewives	2.8 (1.7)
**Lost productivity (patients) ^†**	
Office employees/businessmen	363.2 (576.3)
Laborers	73.4 (102.0)
Housewives‡	46.1 (27.8)
**Lost time (attendants)**	
Office employees/businessmen	3.1 (3.6)
Laborers	3.1 (1. 8)
**Loss of productivity (attendants) #**	
Office employees/businessmen	268.1 (578.5)
Laborers	54.9 (30.1)

• Subsidized consultation and investigation cost is calculated using approximate average of overall respective charges

• ** N = 152, ^N = 305, #N = 67

• † Lost productivity has been estimated by using specific wage rate per hour

• ‡ The wage rate of housewives was estimated using minimum wage rate of Pakistan

### Correlates of cost of care

The mean (SD) direct costs were higher for males compared to females (1,743 vs. 1,555) although the difference was not significant (p = 0.132), however the indirect cost was significantly higher for males compared to females (males: 298; females: 86; p < 0.001). Considering age, the direct cost was marginally higher amongst the older age group compared to younger age groups (p = 0.06). The more educated study subjects expended more on diabetes care and this difference was significant (p < 0.001). By monthly family income, the direct and indirect cost was greater for the higher income families compared to lower income families and this difference was also significant (p < 0.001). Persons of higher SES were spending more as compared to those of lower SES and this difference was found to be significant for both direct (p < 0.001) and indirect (p < 0.001) costs (Table 3).

**Table 3:** Socio-demographic and clinical characteristics correlated with cost of diabetes management among study subjects*

	**Direct cost**		**Indirect cost**	
**Characteristics**	**Mean (SD)**	**p. value**	**Mean (SD)**	**p. value**
**Socio-demographic characteristics**				
**Age**				
20 – 40 years	1477.6 (1555.7)	0.06	153.1 (402.3)	0.142
41 – 50 years	1527.7 (1549.5)		150.9 (362.5)	
51 – 60 years	1814.6 (1530.6)		157.4 (478.4)	
**Sex**				
Male	1742.8 (1569.7)	0.132	298.2 (699.3)	< 0.001
Female	1555.0 (1533.0)		86.0 (111.2)	
**Education**				
Illiterate	746.7 (607.7)	< 0.001	55.2 (44.4)	0.004
Up to primary	1612.8 (1735.2)		162.6 (579.5)	
Secondary to intermediate	1713.4 (1431.7)		155.6 (233.7)	
Graduate and above	2923.9 (1642.7)		300.5 (639.6)	
**Monthly household income**				
Up to 5000	673.8 (575.9)	< 0.001	51.6 (34.5)	< 0.001
5001 – 20000	1354.0 (1314.4)		90.0 (132.0)	
> 20000	2843.0 (1688.4)		332.6 (712.3)	
**Socio-economic status (SES)**				
Low SES	855.0 (618.1)	< 0.001	61.0 (47.7)	< 0.001
Middle SES	1010.0 (1063.5)		78.2 (115.5)	
High SES	2898.1 (1638.9)		300.8 (670.2)	
**Clinical characteristics Duration of DM**				
1–5 years	1412.9 (1425.9)	0.006	167.2 (479.4)	0.824
6–10 years	1680.1 (1517.7)		136.9 (178.7)	
> 10 years	2090.0 (1773.9)		138.6 (442.6)	
**Mode of treatment**				
Lifestyle modification	1302.0 (1163.9)	< 0.001	181.7 (174.3)	0.950
Oral medication	1373.6 (1434.9)		153.0 (424.5)	
Insulin	2574.7 (1546.1)		148.2 (443.3)	
**Co-morbidities**				
No co-morbidity	1226.2 (1327.7)	0.006	165.2 (420.5)	0.583
≥ 1 co-morbidity	1779.0 (1600.3)		149.5 (415.7)	

* All costs are measured in the Pakistani Rupee and for international currency, we considered Rs. 60 per US$.

For clinical characteristics, duration of diabetes, and number of co-morbidities were analyzed with reference to the cost of diabetes care between groups. Mean (SD) direct cost of persons with diabetes with duration of illness between 1–5 years was Rs. [1,413 (1,426)]; 6–10 years was [1,680 (1,518)]; and >10 years was [2,090 (1,774)]; and the cost difference was significantly higher among persons with a longer duration of diabetes (p = 0.006), however the indirect cost difference between these groups was not significant (p = 0.824). The mean (SD) direct cost in persons having no co-morbidity was Rs. [1,226 (1,328)] and those having one or more co-morbidities was [1,779 (1,600)] and this difference was significant (p = 0.006) between groups, while the difference for the indirect cost between these groups was not found significant (p = 0.583). Comparing the mode of treatment with direct cost, we found significant difference (p < 0.001) between those with non-pharmacological lifestyle management to oral medication and insulin, while indirect cost was not significant (p = 0.950) (Table 3).

## Discussion

Diabetes is a costly disease to manage and its cost affects individuals, families, society, healthcare institutions and national productivity. This is the first documented study on the cost of diabetes care conducted in Pakistan. In this study, every possible effort was made to incorporate the majority of cost components associated with diabetes care. Decision-makers are mostly interested in untransformed cost analysis [22] hence we analyzed the untransformed diabetes care cost. However, decision-makers have to allocate resources in the context of scarcity and competition with other sectors (housing, education, etc). Further, it will always be a question as to whether resources are efficiently and effectively allocated. Economic analysis can make an important contribution to the efficient allocation of scarce resources.

This study has produced some important key findings related to the economics of diabetes that may contribute to better healthcare planning. It indicates how much society is spending on diabetes care, which can then be weighted against the cost of implementing prevention programs. Once the diabetes develops then it needs to be controlled to avert the co-morbidities and complications, which can again cause significant cost. Secondly, this study has recognized the cost of diabetes care in relation to different socio-demographic and clinical characteristics. On average one person with diabetes spends Rs. 965 (direct cost only) per month. The overwhelming cost of diabetes threatens to stunt economic growth and undermine the standard of living. Thirdly, it identified the diverse components of cost and the magnitude of the contribution of each component. In some clinics costs (consultation and laboratory investigations) were subsidized, hence a level of subsidy was calculated using approximate average for consultation and laboratory investigations services. This research has been carried out using the societal perspective, which is usually preferred for public health decisions because all the costs including direct, indirect and intangible costs and all resources and benefits, regardless of who pays for or who receives them, are included in the analysis.

The results of this cost of diabetes care study cannot be matched with other studies conducted in developed countries [15,23], mainly because of social and economic differences. Studies on the total cost of diabetes in developing countries are few and of the cost analyses undertaken different techniques have been used, making attempts of comparison problematic.

On socio-demographic factors, higher monthly income and higher education were found positively correlated with increased diabetes cost in this study. Significant correlation with educational status has been reported by other studies conducted in India [24,25]. This may be because of more awareness of disease with higher educational status and that more educated people earn more; hence can afford more for their health. It is well established that the cost of managing diabetes is dominated by old age [26-28], a phenomenon supported by this study.

This study highlights that the direct cost is higher in persons with diabetes of longer duration and one or more co-morbidities and complications. This pattern was also observed in other studies conducted both in developing [24,29] and developed countries [27]. Complications escalate the cost of managing disease and diabetes is associated with many complications, which will ultimately impact on the healthcare system. Likewise longer duration of the disease makes the condition worse which eventually results in higher treatment cost.

In this study, the largest component of total direct healthcare cost was accounted for was medicines followed by laboratory investigations. Excluding hospitalization costs similar findings have been reported in some other studies conducted in developed [14,23,27] as well as developing countries [30]. Overall, the mean direct cost exhibits a positive relationship with the mean income of the family. We found that for persons earning ≤ Rs. 5,000/-, their mean direct cost for treating the diabetes was 18.4% of their mean total income. This is comparable with a study conducted in India, where those on low incomes devote 25% of family income on diabetes care [30].

None of the person with diabetes indicated that their cost is borne by an insurance company or their employer. Almost all the cost is out-of-pocket; from individual or family income. This is not exactly similar to some other studies but Grover and colleagues reported that 95% of cost is met by patients and their families in India [24]. In this part of the world where social protection is virtually non-existent and most expenses are out-of-pocket [31], there is a strong need to develop different health insurance schemes especially for the poorer segment of the population in order to protect their household budget and increase treatment compliance, which will help prevent unnecessary complication(s).

Considering the current prevalence of diabetes in Pakistan, if only the direct cost of diabetes care for out-patient management clinic is calculated, it will be more than Rs. 71 billion for one year. This is 80% greater than the total national health budget (Rs.40 billion) for the current fiscal year. The estimated cost may be termed here as the minimum economic cost of diabetes care in out-patient clinics of Pakistan. These figures would be even higher if hospitalization costs and the costs related to old age (> 60 years) of persons with diabetes were included. Determining the cost of a particular disease is claimed to provide useful information for health policy making and it is argued that such information can help nations to determine research and funding priorities by highlighting areas where inefficiencies may exist and savings could be made [32].

## Conclusion

There is a dearth of disease based economic studies in developing countries and in Pakistan no such study has previously been documented. The estimates here provide information about the use of resources related to diabetes and help to describe the impact that society faces from such a disease. This initial study can lead to further investigations in which cost-effective interventions could be evaluated leading to a potential reduction in the economic burden of diabetes.

In this study, there are some limitations, which should be considered before generalizing the results. First, the inclusion criteria were restricted to out-patients and the 20–60 years age group, also the major cost burden may be attributable to hospitalisations amongst older persons with the disease. Second, the cost of self monitoring of glucose levels was not included. Third, the lost productivity of study subjects and their attendants who were unemployed was not considered.

Nevertheless the information is sufficient enough to make some recommendations. For instance, that easily accessible and affordable healthcare service is required for all persons with diabetes. Availability and affordability of services and drugs can help these people to get the required healthcare services to manage their diabetes. Policy makers need to ascertain the priority of prevention programs at primary healthcare outlets and they also need to ensure the availability of effective patent anti-diabetic drugs at cheaper rates to reduce the medication cost component. The latter must be done without compromising the quality of care. Furthermore, pharmaceutical companies should be encouraged to produce better quality and lower-priced drugs for patients with diabetes.

There is a need to promote more research at a national level on disease-based economic studies to know precisely the social, financial and economic burden of disease in order to inform policy. This would complement international level evidence generated through the WHO-CHOICE project on the costs, impact on population health and cost-effectiveness of various health interventions. More specifically, however, research is needed to identify such cost-effective interventions that will not only lessen the cost of diabetes but improve the overall health status of the persons with diabetes. In addition, healthcare providers need to update themselves on current disease knowledge and best medical practice for better management of diabetes. In particular, this will facilitate preventive strategies and help control the condition through rational prescribing and investigations.

The economic burden of diabetes care is prompting concern throughout the world. Facing the challenges brought about by the epidemic of diabetes, agencies/authorities (NGOs, private and public) will need to work closely if the burden is to be reduced.

## Abbreviations

AKU Aga Khan University

DM Diabetes Mellitus

ERC Ethical Review Committee

NGO Non Governmental Organization

NHSP National Health Survey of Pakistan

Rs. Rupees (Pakistani Currency)

SPSS Statistical Package for Social Sciences

US$ United States Dollars

USA United States of America

## Competing interests

The author(s) declare that they have no competing interests.

## Authors' contributions

LAK was the principal author and contributed to the study concept, design, analysis and interpretation of data.

AKK contributed to the study concept and the interpretation of data.

PC contributed to the analysis of cost data.

All authors read and approved the final manuscript.

## Pre-publication history

The pre-publication history for this paper can be accessed here:



## Supplementary Material

Additional file 1 Questionnaire. The file contains questionnaire carried out for data collection. Click here for file
