# Supplementary material for: Cost of diabetes care in out-patient clinics of Karachi, Pakistan
Source: BMC Health Serv Res. 2007 Nov 21;7:189. doi: 10.1186/1472-6963-7-189 (PMC2206019; doi:10.1186/1472-6963-7-189)
Supplement: Additional file 1 — Questionnaire. The file contains questionnaire carried out for data collection. [file 1472-6963-7-189-S1.doc]

***Questionnaire***

**Title: Cost of diabetes care in out-patient clinics of Karachi, Pakistan**

| ID No. |  |  |  |  |
| --- | --- | --- | --- | --- |

Personal identifier sheet

| 1 | Gender | 2 | Age Yrs. | | DOB (m/d/y) | | | | | | 3 | Marital status |
| --- | --- | --- | --- | --- | --- | --- | --- | --- | --- | --- | --- | --- |
|  |  |  |  |  |  |  |  |  |  |  |  |  |
| 4 | How identified as Diabetic? | 5 | Total time taken  in Interview | | | | | | | | 6 | Date |
|  |  |  |  | | | | | | | |  |  |
| 7 | Language of interview | 8 | Interviewer Name | | | | | | | | 9 | Signature of Interviewer |
|  | Urdu: ____________  Other (specify): _________________ |  |  | | | | | | | |  |  |
|  |  |  |  |  |  |  |  |  |  |  |  |  |
|  |  |  |  |  |  |  |  |  |  |  |  |  |
|  | ***Status*** | | | |  |  |  |  | SUPERVISOR | | | |
|  | Complete: ___________ | | | |  |  |  |  | Checked: __________ | | | |
|  | Incomplete: ___________ | | | |  |  |  |  | Validated: _________ | | | |

***Socio-demographic and general information***

| ID No. |  |  |  |  |
| --- | --- | --- | --- | --- |

| **Q #** | **Question** | **Code** | **Skip pattern** | **Response** |
| --- | --- | --- | --- | --- |
| Q # 1 | What is your current status of education? | Primary …………. 1 |  |  |
|  |  | Secondary ……..... 2 |  |  |
|  |  | Intermediate …...... 3 |  |  |
|  |  | Graduate & above..4 |  |  |
|  |  | Madresah ……..… 5 |  |  |
|  |  | Can read & write…6 |  |  |
|  |  | Illiterate ………….7 |  |  |
|  |  | Other ……………. 0 |  |  |
| Q # 2 | What is your marital status? | Single ………...…. 1 | If 1, go to Q # 4 |  |
|  |  | Married ………..…2 |  |  |
|  |  | Other ……….….... 0 |  |  |
| Q # 3 | How many alive children do you have? | Actual (in numbers) |  |  |
| Q # 4 | What is your employment status? | Office Job ...…… 1 | If 4, Go to Q # 6 |  |
|  |  | Businessman ….... 2 |  |  |
|  |  | Laborer ………… 3 |  |  |
|  |  | Unemployed …… 4 |  |  |
|  |  | Other …….……... 0 |  |  |
| Q # 5 | What is your total monthly income? | Actual (in Rs.) | Now Go to Q # 8 |  |
| Q # 6 | Reason for not being employed? | Jobless ………......1 |  |  |
|  |  | Student ……..……2 |  |  |
|  |  | Housewife …..….. 3 |  |  |
|  |  | Retired …………..4 |  |  |
|  |  | Unable ….……….5 |  |  |
|  |  | Other …. …......…0 |  |  |
| Q # 7 | If unemployed, what are your activities in most of the days of week? | Actual |  |  |
| Q # 8 | What is overall household income? | Actual (in Rs.) |  |  |
| Q # 9 | How many people are supported on this income? | Actual (in numbers) |  |  |
| Q # 10 | Do you own? (in your home) |  |  |  |
|  | (a) Television | Yes………………1 |  |  |
|  |  | No ………………2 |  |  |
|  | (b) Refrigerator | Yes………………1 |  |  |
|  |  | No ………………2 |  |  |
|  | (c) Air Conditioned | Yes………………1 |  |  |
|  |  | No ………………2 |  |  |
|  | (d) Motor-Cycle | Yes………………1 |  |  |
|  |  | No ………………2 |  |  |
|  | (e) Washing Machine | Yes………………1 |  |  |
|  |  | No ………………2 |  |  |
|  | (f) Computer | Yes………………1 |  |  |
|  |  | No ………………2 |  |  |
|  | (g) Car | Yes………………1 |  |  |
|  |  | No ………………2 |  |  |

***Health status and behaviour information***

| ID No. |  |  |  |  |
| --- | --- | --- | --- | --- |

| **Q #** | **Question** | **Code** | **Skip pattern** | **Response** |
| --- | --- | --- | --- | --- |
| Q # 11 | Since how long you have been diagnosed as diabetic? | Actual (Years) |  |  |
| Q # 12 | How often you visit to your doctor due to diabetes? | (Per Year) |  |  |
| Q # 13 | Do you follow the doctor's advice for? |  |  |  |
|  | (a) Consultations | Yes……………. 1 | If 1, Go to Q # 15 |  |
|  |  | No ……………. 2 |  |  |
|  | (b) Laboratory investigations | Yes……………. 1 | If 1, Go to Q # 15 |  |
|  |  | No ……………. 2 |  |  |
|  | (c) Taking medicines | Yes……………. 1 | If 1, Go to Q # 15 |  |
|  |  | No ……………. 2 |  |  |
|  | (c) Dietary Intake | Yes……………. 1 | If 1, Go to Q # 15 |  |
|  |  | No ……………. 2 |  |  |
| Q # 14 | Why you do not follow Doctor's advice? | No knowledge …1 |  |  |
|  |  | No resources ..... 2 |  |  |
|  |  | No time ………. 3 |  |  |
|  |  | Other …….…… 0 |  |  |
| Q # 15 | How do you currently treat the diabetes? | Diet plan/Exercise ..1 |  |  |
|  |  | Diabetes tablets . .. 2 |  |  |
|  |  | Insulin . . . . . . . . . 3 |  |  |
|  |  | Combination . . . . . 4 |  |  |
|  |  | Other . . . . . . . . . .. 0 |  |  |
| Q # 16 | Do you need someone to help in your diabetes care? | Yes ……………. 1 |  |  |
|  |  | No …………….. 2 |  |  |

***Co-morbidities/complications & treatment information***

| ID No. |  |  |  |  |
| --- | --- | --- | --- | --- |

| **Q #** | **Question** | **Code** | **Skip pattern** | **Response** |
| --- | --- | --- | --- | --- |
| Q # 17 | Please tell us about the co-morbidities / complication due to diabetes. Do you have? |  |  |  |
|  | (a) Hypertension | Yes . . . . . . . .. 1 |  |  |
|  |  | No . . . . . . . . . 2 |  |  |
|  | (b) Dyslipidemia | Yes . . . . . . . .. 1 |  |  |
|  |  | No . . . . . . . . . 2 |  |  |
|  | (c) Depression | Yes . . . . . . . .. 1 |  |  |
|  |  | No . . . . . . . . . 2 |  |  |
|  | (d) Heart disease | Yes . . . . . . . .. 1 |  |  |
|  |  | No . . . . . . . . . 2 |  |  |
|  | (e) Retinopathy | Yes . . . . . . . .. 1 |  |  |
|  |  | No . . . . . . . . . 2 |  |  |
|  | (f) Neuropathy | Yes . . . . . . . .. 1 |  |  |
|  |  | No . . . . . . . . . 2 |  |  |
|  | (g) Nephropathy | Yes . . . . . . . .. 1 |  |  |
|  |  | No . . . . . . . . . 2 |  |  |
|  | (h) Other ________________ | Yes . . . . . . . .. 1 |  |  |
|  |  | No . . . . . . . . . 2 |  |  |
| Q # 18 | Do you use medication for? |  |  |  |
|  | (a) Hypertension | Yes . . . . . . . .. 1 |  |  |
|  |  | No . . . . . . . . . 2 |  |  |
|  | (b) Dyslipidemia | Yes . . . . . . . .. 1 |  |  |
|  |  | No . . . . . . . . . 2 |  |  |
|  | (c) Depression | Yes . . . . . . . .. 1 |  |  |
|  |  | No . . . . . . . . . 2 |  |  |
|  | (d) Heart disease | Yes . . . . . . . .. 1 |  |  |
|  |  | No . . . . . . . . . 2 |  |  |
|  | (e) Retinopathy | Yes . . . . . . . .. 1 |  |  |
|  |  | No . . . . . . . . . 2 |  |  |
|  | (f) Neuropathy | Yes . . . . . . . .. 1 |  |  |
|  |  | No . . . . . . . . . 2 |  |  |
|  | (g) Nephropathy | Yes . . . . . . . .. 1 |  |  |
|  |  | No . . . . . . . . . 2 |  |  |
|  | (h) Other ________________ | Yes . . . . . . . .. 1 |  |  |
|  |  | No . . . . . . . . . 2 |  |  |

***Treatment regime information***

| ID No. |  |  |  |  |
| --- | --- | --- | --- | --- |

| **Q #** | **Question** | **Dose** | **Frequency** | **Expenses** |
| --- | --- | --- | --- | --- |
| Q # 19 | Which medicines you use for diabetes and other co-morbidities / complications in last month? |  |  |  |
|  | (a) |  |  |  |
|  | (b) |  |  |  |
|  | (c) |  |  |  |
|  | (d) |  |  |  |
|  | (e) |  |  |  |
|  | (f) |  |  |  |
|  | (g) |  |  |  |
|  | (h) |  |  |  |
|  | (i) |  |  |  |
|  | (j) |  |  |  |
|  | (k) |  |  |  |
|  | (l) |  |  |  |
|  | (m) |  |  |  |
|  | (n) |  |  |  |
| Q # 20 | Do you use insulin to treat your diabetes? | Yes . . . . . . . .. 1 | If 2, go to Q # 22 |  |
|  |  | No . . . . . . . . . 2 |  |  |
| Q # 21 | Use of insulin? | **Dose** | **Frequency** | **Expenses** |
|  |  |  |  |  |

***Diabetes cost information***

| ID No. |  |  |  |  |
| --- | --- | --- | --- | --- |

| **Q #** | **Question** | **Code** | **Skip pattern** | **Response** |
| --- | --- | --- | --- | --- |
| Q # 22 | How much money you spent for? | In current visit  (In rupees) |  |  |
|  | (a) consultation | Actual |  |  |
|  | (b) lab tests | Actual |  |  |
|  | (c) medicines | Actual |  |  |
|  | (d) travel cost | Actual |  |  |
|  | (e) food cost | Actual |  |  |
|  | (f) other health care cost | Actual |  |  |
| Q # 23 | How much time you spent during? | In current visit (in minutes) |  |  |
|  | (a) Travelling to clinic | Actual |  |  |
|  | (b) Waiting in clinic | Actual |  |  |
|  | (c) Consultation | Actual |  |  |
| Q # 24 | For how long you take leave from your employer for each visit? | 0 hours …...….. 1 |  |  |
|  |  | 1-2 hours ……. 2 |  |  |
|  |  | 2-4 hours ……. 3 |  |  |
|  |  | 1 day ..………. 4 |  |  |
|  |  | Other ............... 0 |  |  |
| Q # 25 | Does anyone accompany you to the clinic? | Yes…………… 1 | If 2, go to Q # 28 |  |
|  |  | No …………… 2 |  |  |
| Q # 26 | What is his / her job? | Office Job ........ 1 | If 4, go to Q # 28 |  |
|  |  | Businessman ….2 |  |  |
|  |  | Laborer ……….3 |  |  |
|  |  | Unemployed … 4 |  |  |
|  |  | Other …….…... 0 |  |  |
| Q # 27 | What is his/her monthly income? | Actual |  |  |
| Q # 28 | Because of cost, do you: |  |  |  |
|  | (a) Skip a pill or insulin shot? | Yes………………1 |  |  |
|  |  | No ………………2 |  |  |
|  | (b) Skip checking your blood sugar? | Yes………………1 |  |  |
|  |  | No ………………2 |  |  |
|  | (c) Skip consultation? | Yes………………1 |  |  |
|  |  | No ………………2 |  |  |
|  | (d) Other __________________________ | Yes………………1 |  |  |
|  |  | No ………………2 |  |  |
| Q # 29 | Who is responsible for the finances of your diabetes treatment? | Self ….………….1 |  |  |
|  |  | Spouse ………….2 |  |  |
|  |  | Parent ………..... 3 |  |  |
|  |  | Brother/sister ….. 4 |  |  |
|  |  | Son / Daughter ... 5 |  |  |
|  |  | Employer ……… 6 |  |  |
|  |  | Health insurance..7 |  |  |
|  |  | Other ………...…0 |  |  |
| Q # 30 | Any extra information on expenses incurred due to diabetes? | Actual |  |  |
